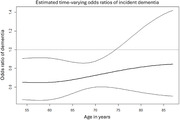# The Associations of Physical Activity from Midlife to Late Life with Incident Dementia: The Health and Retirement Study, 1996‐2020

**DOI:** 10.1002/alz.088256

**Published:** 2025-01-09

**Authors:** Yanan Zhang, Chih‐Hsiang Yang, Anwar T Merchant, Jingkai Wei

**Affiliations:** ^1^ University of South Carolina, Columbia, SC USA

## Abstract

**Background:**

While physical activity is found to be associated with a lower risk of dementia in numerous studies, less is known whether this association varies over time. We aim to examine the potential time‐varying associations of physical activity with risk of dementia from midlife to late life.

**Method:**

Participants aged 54 to 64 years of the Health Retirement Study (HRS) in 1996 were included. Physical activity was measured using self‐report questionnaires. Physically active was defined as having vigorous activity ≥3 times a week during 1996‐2002, and as having vigorous activity >1 time a week during 2004‐2020. Ascertainment of dementia was based on the Langa‐Weir Classification of Cognitive Function (range 0‐27), which included immediate and delayed recall items, the serial 7s, and backward counting. A score <6 was considered having dementia. Participants were followed up until the occurrence of dementia, death/lost to follow‐up, or the last interview in 2020, whichever came first.

A time‐varying effect logistic regression was fit to examine the trend of the association between physical activity and dementia across ages. Regression covariates includes time‐invariant variables (age at baseline, sex, race/ethnicity, and education) and time‐variant variables [BMI (Kg/m^2^), current smoking status (Yes/No), number of days/week drinks, hypertension (Yes/No), diabetes (Yes/No), cardiovascular diseases (Yes if reporting any of heart problems or stroke), and cancers (Yes/No)]. Missing values were imputed by the values from the previous interview. To check the impact of revised definitions of physical activity, a sensitivity analysis was also conducted.

**Result:**

A total of 7,001 participants were included in the analysis. During an average follow‐up period of 15.1±7.8 years, 736 cases of dementia occurred. Being physically active in midlife was associated with a lower risk of dementia. The association was not static and became attenuated with aging, and no significant association was observed after the age of 75.

**Conclusion:**

The association of physical activity with incident dementia attenuated throughout the progress of aging. While the possibility of reverse causality needs to be further explored, our results may suggest that physical activity may be initiated at an earlier stage of life for risk reduction of dementia.